# Comparative Analysis of Lineage Structure, Cellulose Locus Context, and Mobilome Diversity Across Complete *Komagataeibacter* Genomes

**DOI:** 10.3390/microorganisms14030653

**Published:** 2026-03-13

**Authors:** Mustafa Guzel

**Affiliations:** Department of Food Engineering, Hitit University, Corum 19030, Türkiye; mustafaguzel@hitit.edu.tr

**Keywords:** *Komagataeibacter*, bacterial cellulose, pangenome, plasmid conservation, mobile genetic elements

## Abstract

*Komagataeibacter* strains are important bacterial cellulose producers, yet closely related isolates can differ in cellulose yield, pellicle properties, and genetic stability during propagation. Such variability suggests that lineage structure and mobile genetic elements both contribute to strain-level genomic divergence. Here, complete genome comparisons were used to integrate vertical relatedness, gene-content structure, cellulose-associated signatures, and mobilome heterogeneity across 22 closed *Komagataeibacter* assemblies. A maximum likelihood phylogeny inferred from 642 single copy core genes provided the lineage scaffold. An anvi’o pangenome analysis defined a constant core gene cluster component across genomes and a noncore fraction that accounted for most of the genome differences in gene content. Targeted features linked to cellulose biosynthesis and local c-di-GMP-associated context were extracted from each genome. These features captured differences in bcs neighborhood composition and the presence of nearby GGDEF and EAL domain signals. The resulting feature matrix was projected by principal component analysis to summarize between-genome variation. Mobilome profiles were strongly strain dependent. Plasmid homology clustering identified 12 clusters comprising 36 plasmids from 13 genomes, including two dominant clusters of seven and six plasmids. Mash-based distance summaries further distinguished clusters consistent with conserved backbones from clusters consistent with heterogeneous, module-driven relationships. Prophage sequences, assessed as VIBRANT-predicted regions, were widespread but sparse per genome and dominated by medium length fragments. Insertion sequence burden ranged from 50 to 181 elements per genome, indicating substantial differences in transposition-associated sequence content. Pairwise association tests did not support robust cross module covariation beyond expected relationships among pangenome composition metrics at the current sampling depth. Overall, these results provide a complete genome reference framework linking lineage structure and mobilome heterogeneity, and they define reusable resources for comparative studies in bacterial cellulose biotechnology.

## 1. Introduction

Bacterial cellulose (BC) is a high-value microbial biopolymer used in medical, pharmaceutical, and food-related applications. Its utility derives from high water-holding capacity, a nanofibrillar architecture, favorable mechanical properties, and biocompatibility [1]. Despite expanding application interest, production cost remains a major constraint on broader industrial adoption, and strain selection and optimization are central levers for improving process economics [2]. Among acetic acid bacteria, *Komagataeibacter* is widely regarded as a major group of efficient BC producers and has been refined taxonomically through genome-informed revisions relative to closely related acetic acid bacterial lineages, including the reassignment of several species to *Novacetimonas* [3,4]. Consistent with this ecology, *Komagataeibacter* strains have been reported from acidic, fermentation, and plant-associated habitats, including vinegar-related environments, kombucha, fruits and fruit juices, and nata de coco systems [3,5]. This combination of material relevance and ecological breadth motivates genome-resolved comparisons aimed at explaining why closely related BC-producing strains differ in cellulose-related genomic signatures and associated adaptive potential.

Cellulose production in *Komagataeibacter* is phenotypically heterogeneous across strains and species. Variation has been reported in carbon source utilization, nanocellulose yield and production rate, pellicle architecture, and strain stability. Genome-informed taxonomic revisions further complicate cross-study comparisons because historical strain labels do not always map cleanly onto current species boundaries [6]. Comparative inference is therefore best anchored in a genome-resolved lineage structure rather than inferred from legacy nomenclature alone [4,6].

Beyond a reliable taxonomic framework, the genetic determinants that shape strain level differences in bacterial cellulose production need to be defined. At the mechanistic level, bacterial cellulose synthesis is mediated by cellulose synthase systems encoded by bcs loci. A canonical four-gene bcsABCD operon was first characterized in the historical *Acetobacter* and *Gluconacetobacter xylinus* lineage, which is now classified within *Komagataeibacter* [7]. BcsA and BcsB form the catalytic core that supports the polymerization and translocation of the glucan chain. BcsC and BcsD contribute to export and extracellular assembly and are required for maximal cellulose production in vivo [7]. Cellulose synthesis is regulated by the second messenger cyclic-di-GMP, which activates the synthase through binding to the PilZ domain of BcsA [8,9]. Cellular cyclic-di-GMP levels are set by opposing enzyme classes. Diguanylate cyclases typically carry GGDEF domains, whereas phosphodiesterases commonly carry EAL or HD GYP domains [9,10]. *Komagataeibacter* genomes often encode multiple cellulose synthase operons and diverse bcs architectures. Differences in operon composition and organization have been described, and multiple distinct operons can coexist within a single genome [5,11]. Such modularity provides a plausible genomic basis for variation in cellulose-associated features across strains and species [12]. In industrial settings, performance is also shaped by genetic stability during propagation and repeated fermentation cycles. In cellulose-producing acetic acid bacteria, insertion sequence activity has been linked to a loss of cellulose production through disruptive insertions in cellulose biosynthesis loci, including insertions affecting bcsA that yield stable cellulose negative variants [13,14].

While variation in core operon architecture contributes to cellulose-related diversity, additional plasticity can arise through horizontal acquisition and genome remodeling. Mobile genetic elements are key drivers of bacterial genome plasticity because they mediate horizontal transfer and promote gene gain, gene loss, and structural rearrangement [15]. Plasmids are particularly relevant because they can couple mobility functions with accessory traits that are conditionally beneficial, which allows for rapid shifts in ecological capacity without requiring a deep divergence of the core genome [16]. Temperate phages can also contribute to diversification. When integrated as prophages, they can alter host phenotypes through lysogenic conversion, although fitness effects are environment-dependent and not universal [17,18]. In parallel, insertion sequences can restructure genomes through the disruption of coding sequences and promotion of rearrangements, thereby generating strain-specific signatures even when recognizable cargo enrichment is not apparent under strict definitions [15,19]. These mechanisms are directly relevant to *Komagataeibacter*. Comparative genome studies have shown that closely related strains can differ substantially in accessory gene content and in the distribution of repeated and mobile element-associated sequences [5]. Recent complete genome work further emphasizes that complete assemblies remain limited for the genus, which constrains the resolution of plasmid inventories and other mobilome components [20]. From a biotechnology perspective, this motivates explicit mobilome-resolved comparisons. Strain-level differences in robustness and production-relevant traits are expected to reflect both lineage-specific cellulose and regulatory architecture and differences in mobilome-encoded accessory potential that is unevenly distributed across genomes [15,16].

Despite the increasing availability of genome sequences for cellulose-producing acetic acid bacteria, the genomic basis of strain-level divergence within *Komagataeibacter* remains only partly resolved. Prior studies have reported diversity in cellulose-related loci and broader gene content [21]. However, lineage structure, cellulose-locus neighborhood features with nearby c-di-GMP-associated annotations, and mobilome architecture with plasmid-sharing patterns have often been examined separately. This limits the interpretation of plasmid family structure, prophage-associated regions, and IS burden in a single lineage-resolved comparative frame [15,22]. Complete assemblies were used because plasmid inventories, insertion sequence burden, and prophage-associated regions are difficult to summarize reliably from fragmented draft assemblies.

Here, a curated set of complete *Komagataeibacter* genomes was used to address this gap. Lineage relationships were defined using core genome phylogenomics. Cellulose-related and c-di-GMP-associated features were summarized using a targeted signature-based approach. Mobilome components were characterized by an emphasis on plasmid repertoire diversity, plasmid family conservation, and complementary summaries of prophage-associated regions and insertion sequence burden. This design enables mobilome variation to be interpreted against a stable lineage scaffold while retaining a direct link to cellulose-associated genomic features relevant to biotechnology. These analyses provide a lineage-resolved comparative framework and reusable genomic resources, and they are not interpreted as direct mechanistic tests of cellulose yield determinants.

## 2. Materials and Methods

### 2.1. Genome Dataset and Curation

Complete *Komagataeibacter* genome assemblies were retrieved from RefSeq and curated into a fixed manifest prior to analysis. The initial dataset comprised 22 complete assemblies, each containing a chromosome and associated plasmid sequences. Complete genomes were selected to support mobilome analyses that depend on accurate plasmid reconstruction, reliable IS detection, and interpretable prophage-region coordinates. For mobilome-focused analyses, a nonredundant subset of 18 genomes was used. Four clonal or derivative assemblies were excluded to avoid the inflation of signals from near-identical genomes. A metadata file was maintained to record genome identifiers, species and strain labels, and inclusion status. This ensured that all analyses used a consistent frozen dataset. A unified metadata framework was used to cross-reference NCBI accessions with strain designations, assembly metrics, and source environments (Table S1).

### 2.2. Core Genome Reconstruction and Phylogenomic Inference

Single-copy core genes were defined using anvi’o pangenome outputs and extracted as a concatenated alignment for phylogenomic reconstruction. The anvi’o platform was used for the organization, inspection, and export of the high-homology core gene set, following the anvi’o framework [23]. A maximum likelihood phylogeny was inferred from the concatenated single copy core alignment using IQ-TREE2, including ultrafast bootstrap support estimation [24]. Branch support values were extracted from the IQ-TREE support tree output and mapped to the final tree visualization. Pairwise average nucleotide identity was computed using pyANI [25]. ANI identity and alignment coverage matrices were generated for the quality control of pairwise comparisons. These matrices were used to summarize genome relatedness and to generate ANI-derived Newick trees for comparison with the core genome phylogeny.

The core genome tree was visualized and annotated using Interactive Tree Of Life (iTOL). iTOL was used to apply consistent species and strain labels, colored taxonomy bands, and additional annotation tracks for selected features [26].

### 2.3. Pangenome Inference and Binning into Core and Accessory Components

Pangenome analysis was performed using anvi’o pangenomics [23]. Gene clusters were binned into core-associated and non-core components using anvi’o bin definitions that separate high-homology core, single-copy core, multi-copy core, and additional core bins, along with accessory and singleton categories. Outputs included per-genome bin counts and plasticity metrics, as well as gene-cluster to bin mappings. These outputs were used to summarize invariance in the core-associated component and quantify heterogeneity in accessory and singleton content across genomes.

### 2.4. Cellulose and Regulatory “Richness” Module

A targeted module was constructed to quantify variation in cellulose synthesis features and c-di-GMP associated signals across genomes. The target set included cellulose synthase and associated bcs genes and c-di-GMP-signaling proteins. For targets occurring in multiple copies or in multiple bcs operon configurations, a standardized selection strategy was used to support cross-genome comparability. Target proteins were identified per genome. Representative sequences were selected per target using a best-hit strategy with local context checks where applicable. To standardize locus context comparisons, a single-anchor contig was selected per genome for the bcs neighborhood analysis. The anchor contig was defined as the contig on which bcsA and bcsZ co localized. When this criterion was met, bcsQ was also selected from the same contig when present. Genomes that did not meet the co-localization criterion were flagged and excluded from anchor-based window summaries. Local GGDEF and EAL domain signals were summarized within 50 kb of the anchor locus. Similarity was summarized relative to a medoid reference when appropriate. A similarity matrix was generated across genomes and reduced by principal component analysis. PC scores were used for the richness PCA visualization. To support the interpretation of multicopy architectures, bcs operon architecture features were summarized in a binary table. bcsA multicopy patterns were summarized separately as an auxiliary analysis.

### 2.5. Insertion Sequence Detection and IS Burden Quantification

Insertion sequences were identified using ISEScan 1.7.2.2, which detects IS elements with curated transposase profile HMMs and generates genome-wide IS annotations as described by Ref. [27]. IS burden was summarized per genome using two primary metrics: total IS count and cumulative IS sequence length. These summaries were used to quantify mobilome heterogeneity across the full 22-genome set. Additional proximity analyses were performed by computing distances between genes and nearby IS elements to assess whether curated functional categories were enriched near IS boundaries. Proximity analysis did not reveal a statistically significant enrichment of biotechnology-relevant functional categories within the immediate vicinity of identified IS elements under the parameters used.

### 2.6. Prophage Region Prediction and Characterization

Prophage region summaries were restricted to genomes meeting the contiguity criteria used for region delineation, resulting in a 16-genome subset. Prophage regions were predicted using VIBRANT v1.2.1 [28]. VIBRANT outputs represent predicted prophage regions or fragments. These results were therefore treated as predicted regions rather than validated intact prophages. Region inventories were generated from renamed VIBRANT region FASTA outputs. Region count and total predicted prophage region length were summarized per genome. Region length distributions were summarized across the dataset. Where cross-genome similarity comparisons were performed, region-to-region comparisons were reported as the best cross-genome hits and pairwise similarity tables. Prophage-associated sequence was additionally assessed with Cenote-Taker 3 as an independent viral sequence discovery and annotation workflow. Cenote-Taker 3 v3.4.4 was run on each genome assembly FASTA using default settings [29]. For each genome, Cenote-Taker 3 outputs were summarized as the number of predicted viral regions and the total length of the predicted viral sequence. These per-genome burden summaries were compared with the VIBRANT summaries.

### 2.7. Plasmid Identification, Clustering, and Validation

Plasmid sequences from each complete assembly were compiled into a plasmid sequence catalog. Plasmid mobility features were assessed using MOB-suite [30]. Plasmid sequences were clustered into homology groups to summarize plasmid sharing across genomes and species labels. Cluster distributions were summarized as a presence absence matrix across genomes and used for a genome by cluster heatmap aligned to taxonomy.

Within-cluster Mash distances were computed to evaluate whether clusters were consistent with conserved plasmid backbones or more heterogeneous module sharing. Mash distances were estimated using MinHash-based sketching [31]. Within each plasmid cluster, all-by-all Mash distances were calculated and summarized as the median, minimum, and maximum within-cluster distance. Clusters were categorized as highly conserved when the within-cluster median Mash distance was below 0.02, as moderately conserved when the median was between 0.02 and 0.05, and as mosaic-like when the median exceeded 0.05. These labels are used as within-dataset descriptors of distance dispersion and do not represent externally benchmarked plasmid-family definitions.

### 2.8. Visualization, Figure Export, and Reproducible Assets

All plots were generated in R. Figures were produced using ggplot2 [32] and exported as high-resolution TIFF and PDF files. Styling was standardized to support taxonomy-aware labeling and consistent readability. Final analysis inputs and outputs were staged in a reproducible asset directory.

## 3. Results

Twenty-two complete *Komagataeibacter* assemblies were curated. Each assembly included a chromosome and plasmid sequences when present. Ten named species were represented. Four assemblies were treated as clonal or derivative for mobilome conservation analyses and were excluded from those comparisons. These were *K. rhaeticus* ENS 9a1a with accession GCF_011611525.1, *K. rhaeticus* ENS9b with accession GCF_014725815.1, *K. sucrofermentans* JML KO23 with accession GCF_040581375.1, and *K. sucrofermentans* JML 2321 with accession GCF_040581385.1. The resulting nonredundant set comprised 18 genomes and was used for the main comparative mobilome analyses.

Within this 18-genome set, single genomes were available for *K. medellinensis*, *K. europaeus*, and *K. diospyri*. Three species were represented by single genomes in this dataset, and patterns involving these taxa are interpreted as dataset-level observations rather than species-level generalizations. Two or three strains were available for the remaining taxa, as summarized in Table 1. Genome size in the nonredundant set ranged from 3.44 to 4.24 Mb, with a median of 3.76 Mb. Isolation metadata indicated fermentation and plant-associated origins, including kombucha-associated sources, vinegar-associated sources, and fruit or plant-associated sources. One genome was linked to an insect-associated isolation source. Three genomes lacked source annotations. These summaries are provided in Table 1.

### 3.1. Core Genome Phylogenomics

A core genome phylogeny was inferred from a concatenated alignment of single-copy core genes extracted with anvi’o. The alignment comprised 642 single-copy core genes and was analyzed using maximum likelihood inference with IQ-TREE. The resulting tree provided the lineage scaffold used for interpreting genome content and mobilome variation across the dataset.

**Figure 1:**
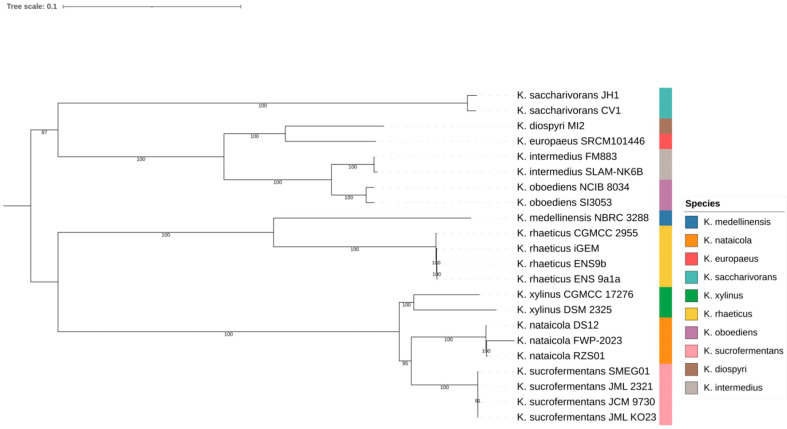
Core-genome phylogeny of *Komagataeibacter* genomes. Maximum-likelihood tree inferred from concatenated single-copy core genes; colors indicate species.

Support values across internal nodes ranged from 87 to 100 in the inferred tree. Multiple species formed compact within-species clusters, as shown in Figure 1. These included *K. nataicola* strains RZS01, DS12, and FWP 2023; *K. saccharivorans* strains CV1 and JH1; *K. xylinus* strains DSM 2325 and CGMCC 17276; *K. oboediens* strains SI3053 and NCIB 8034; and *K. intermedius* strains SLAM NK6B and FM883. Several named species were separated by short internal branches, consistent with close relatedness in the core genome backbone. One example was the placement of *K. europaeus* SRCM101446 adjacent to *K. diospyri* MI2, shown in Figure 1. This lineage scaffold was used to interpret genome content variation, beginning with genus wide pangenome structure.

### 3.2. Pangenome Composition

**Figure 2:**
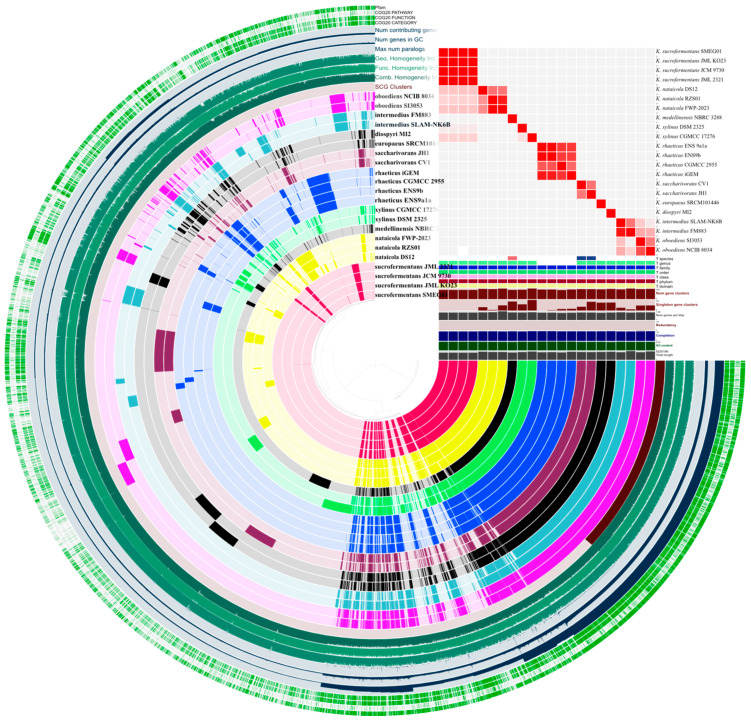
Pangenome structure and genome relatedness across *Komagataeibacter*. An anvi’o pangenome view showing gene-cluster distribution across genomes (**left**) alongside the ANI identity heatmap and genome metadata tracks (**right**).

An anvi’o pangenome analysis was used to quantify the gene content structure across the genome dataset (Figure 2). The pangenome comprised 8682 gene clusters. A core associated set of 1776 gene clusters was identified, and 6906 clusters were assigned to the noncore fraction.

The noncore fraction varied across genomes. Total gene cluster counts ranged from 2896 to 3401 per genome, with a median of 3128. Accessory gene clusters ranged from 903 to 1563 per genome, with a median of 1251. Singleton gene clusters showed the widest dispersion, with a median of 95 and a maximum of 326. Unassigned clusters were rare across genomes and remained below 10 per genome. Full per-genome summaries and bin level breakdowns are provided in Table S2.

The largest per-genome gene cluster count and the largest singleton count were observed in *K. xylinus* CGMCC 17276. The lowest total cluster count was observed in *K. rhaeticus* CGMCC 2955. Overall, these results indicate a stable core gene cluster component and a noncore fraction that accounts for most between genome variability in gene content (Figure 2). Given that genome variability was concentrated in the noncore fraction, subsequent analyses focused on trait-relevant loci and local regulatory neighborhoods linked to cellulose synthesis.

### 3.3. Cellulose and Regulatory Signatures

**Figure 3:**
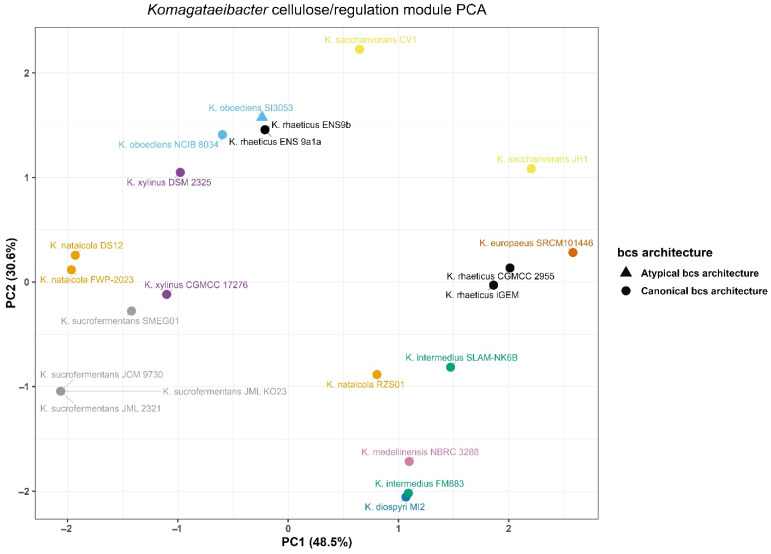
Principal component analysis of cellulose biosynthesis locus architecture across *Komagataeibacter* genomes.

Variation in cellulose-related loci and nearby c-di-GMP-associated signals was summarized using the richness module and its PCA representation (Figure 3). The analysis was structured to enable cross-genome comparison in the presence of multi-copy targets. For each genome, a single anchor locus was selected to represent a comparable bcs neighborhood. Features were then derived relative to this anchor.

Across the 18-genome set, bcsA and bcsZ were located on the same contig in 17 genomes. In these same 17 genomes, bcsQ was also selected on the anchor contig. The remaining genome, *K. oboediens* SI3053, did not meet the anchor contig criteria. Multi-copy bcsA signals were frequently detected in the target searches. Most genomes concentrated these hits on a single contig. Three genomes showed dispersed bcsA hits across multiple contigs. These were *K. oboediens* SI3053, *K. saccharivorans* CV1, and *K. saccharivorans* JH1.

The local c-di-GMP-associated context around the anchor also varied among genomes. A GGDEF domain signal within 50 kb of the anchor was detected in 14 genomes. An EAL domain signal within 50 kb of the anchor was also detected in 14 genomes. Four genomes lacked both signals within this window. These were *K. saccharivorans* CV1, *K. saccharivorans* JH1, *K. oboediens* SI3053, and *K. intermedius* FM883.

Principal component analysis of richness features showed broad separation across genomes in the resulting PCA space (Figure 3). Richness PC1 ranged from −2.112 to 2.644. Richness PC2 ranged from −2.102 to 2.278. Several genomes occupied positions near the extremes of the projection. *K. europaeus* SRCM101446 had the highest richness PC1 value. *K. sucrofermentans* JCM 9730 and two *K. nataicola* genomes showed the lowest richness PC1 values. The most negative richness PC2 values were observed for *K. diospyri* MI2 and *K. intermedius* FM883. The most positive richness PC2 values were observed for *K. saccharivorans* CV1 and both *K. oboediens* genomes.

### 3.4. Plasmid Repertoires and Conservation

Plasmid sharing across genomes was summarized by clustering plasmid sequences into homology groups and mapping cluster membership across strains. Twelve plasmid homology clusters were identified. These clusters comprised 36 plasmids from 13 genomes. Plasmid clustering therefore captured sharing among a subset of strains rather than across all genomes included in the study.

**Figure 4:**
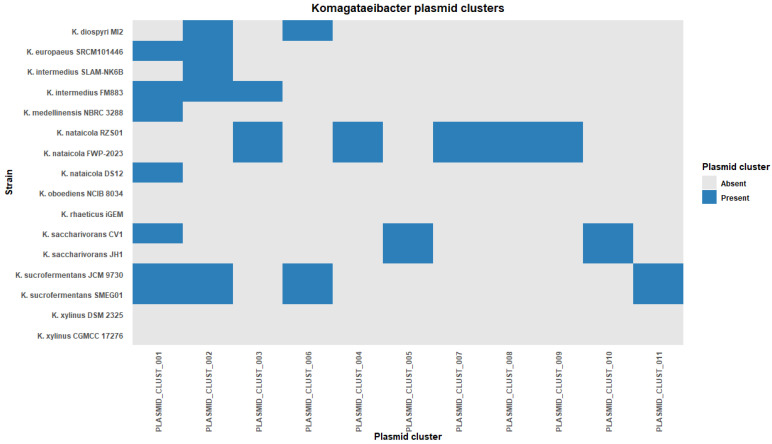
Plasmid homology cluster distribution across *Komagataeibacter* genomes.

Cluster sizes were uneven. Cluster 1 contained seven plasmids and Cluster 2 contained six plasmids. Together, these two clusters accounted for 13 of 36 clustered plasmids. The remaining clusters contained between one and three plasmids each (Table S3). A genome-by-cluster presence/absence heatmap showed a patchy distribution of clusters across strains (Figure 4). Several clusters spanned labels of multiple species. Cluster 1 was detected in six species and Cluster 2 was detected in four species. Cluster 3 and Cluster 6 were each detected in two species. In contrast, eight clusters were restricted to a single species label in this dataset (Figure 4). This pattern indicates that some plasmid clusters are shared across species labels, whereas others are limited to a narrower host background at the scale captured here.

Within-cluster similarity was evaluated using Mash-based distance summaries to distinguish clusters consistent with conserved plasmid backbones from clusters consistent with more heterogeneous relationships. Six clusters were classified as highly conserved, five as moderately conserved, and one as mosaic-like based on within-cluster distance distributions (Table S3). Median Mash distances spanned approximately 0.001 to 0.052 across clusters. The number of within-cluster pairs ranged from 1 to 21. The mosaic-like cluster corresponded to Cluster 1 and showed the broadest within-cluster dispersion, which is consistent with heterogeneous whole-plasmid relationships among members of that cluster (Table S4). As an orthogonal validation, within-cluster gene content concordance was summarized from Prokka annotations using pairwise Jaccard overlap of predicted CDS product sets. This analysis showed high concordance for several small clusters and lower median concordance with broader dispersion for Cluster 1 (Table S3). These validation results clarify the interpretation of the presence/absence heatmap by distinguishing clusters that likely represent conserved backbones from clusters that may be unified by shared sequence modules despite heterogeneous overall similarity. MOB-suite mobility predictions were summarized for the plasmid subset. Predicted conjugative and mobilizable plasmids were present at low abundance across genomes, with a maximum of three per genome. Rep-only non-mobilizable plasmids were limited to zero or one per genome. MOB-suite also returned “unknown” classifications in some genomes, indicating that mobility could not be inferred from available signatures. Relaxase types were reported where detected and were dominated by MOBQ, MOBF, and MOBP annotations (Table S10).

Plasmid-associated annotations were then summarized to describe the distribution of predicted functional categories across genomes, without assigning phenotypic effects. After establishing plasmid cluster distributions and validation categories, plasmid gene annotations were summarized to compare predicted functional categories across genomes.

### 3.5. Plasmid-Associated Biotech and Fitness-Related Cargo

Plasmid-associated genes were screened for annotation categories used as comparative descriptors of plasmid gene content across genomes. These annotations represent predicted functions. They were used to summarize strain-resolved differences in plasmid-encoded gene repertoires without assigning phenotypic effects. Genome-level summaries are provided in Table S5 and Figure S1.

Total plasmid-associated gene counts ranged from 12 to 201 per genome, with a median of 110. Mobility-associated genes comprised a large fraction of these annotations in several genomes. Mobility-associated gene counts ranged from 7 to 153 per genome, with a median of 88. Non-mobility annotated categories ranged from 4 to 48 per genome, with a median of 21. Variation in total plasmid-associated gene counts therefore primarily reflected differences in the abundance of mobility-associated genes, whereas non-mobility categories contributed to a smaller but variable component across genomes.

Marked between genome differences were observed in these summaries. *K. intermedius* FM883 showed the highest total count, with 201 plasmid-associated genes and 153 mobility-associated genes. In the same genome, defense-annotated genes reached 29 and acid and pH-annotated genes reached 6. At the lower end of the distribution, *K. intermedius* SLAM NK6B had twelve plasmid-associated genes and seven mobility-associated genes. This contrast indicates that plasmid-associated annotation profiles can differ strongly between strains that share a species label in this dataset. Metal stress-annotated genes ranged up to 10 per genome, with the maximum observed in *K. xylinus*. CGMCC 17276 (Table S5, Figure S1).

### 3.6. Prophage Region Burden and Length Distributions

The prophage region was summarized for 16 genomes after excluding clonal or derivative assemblies and two additional fragmented assemblies that did not meet contiguity criteria. The prophage-associated sequence was summarized using VIBRANT predicted prophage regions. This analysis was restricted to the 16-genome subset for which region inventories and length summaries were generated (Table S6). These outputs represent predicted regions and were therefore treated as putative prophage-associated fragments rather than validated intact prophages. Region counts and lengths are reported as comparative summaries of VIBRANT and Cenote-Taker 3 detected prophage-associated sequences.

**Figure 5:**
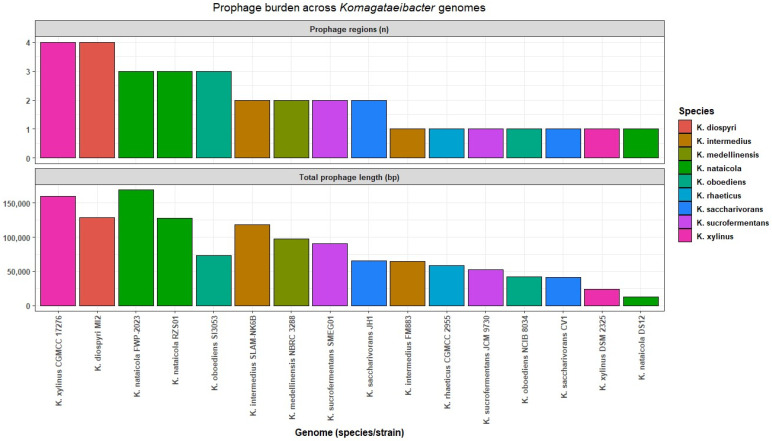
Prophage burden across *Komagataeibacter* genomes.

Across the 16 genomes, 32 predicted regions were identified. Region counts ranged from one to four per genome, with a median of two. The total predicted prophage region sequence per genome ranged from 12,461 bp to 168,872 bp, with a median of 69,219 bp. The largest total predicted prophage region sequence was observed in *K. nataicola* FWP 2023, which carried three regions summing up to 168,872 bp. The highest region count was observed in *K. xylinus* CGMCC 17276, which carried four regions summing up to 159,501 bp. The lowest total predicted prophage region sequence was observed in *K. nataicola* DS12, which carried one region of 12,461 bp. Genomes with similar region counts differed in total predicted prophage region sequence, indicating that genome variation reflected both region number and region length (Table S6, Figure 5). Cenote-Taker 3 produced a broadly concordant per-genome burden pattern by rank order, supporting the interpretation that prophage-associated sequence is widespread but sparse across genomes at the scale captured here (Table S6).

Region lengths ranged from 10,080 bp to 70,900 bp. The median length was 41,913 bp and the interquartile range spanned 32,383 bp to 52,529 bp (Figure S2). These summaries show that the predicted prophage region signal was dominated by medium-length regions in this dataset. Consistent with the VIBRANT summaries, Cenote-Taker 3 detections were treated as predicted regions rather than validated intact prophages.

### 3.7. Insertion Sequence Burden

**Figure 6:**
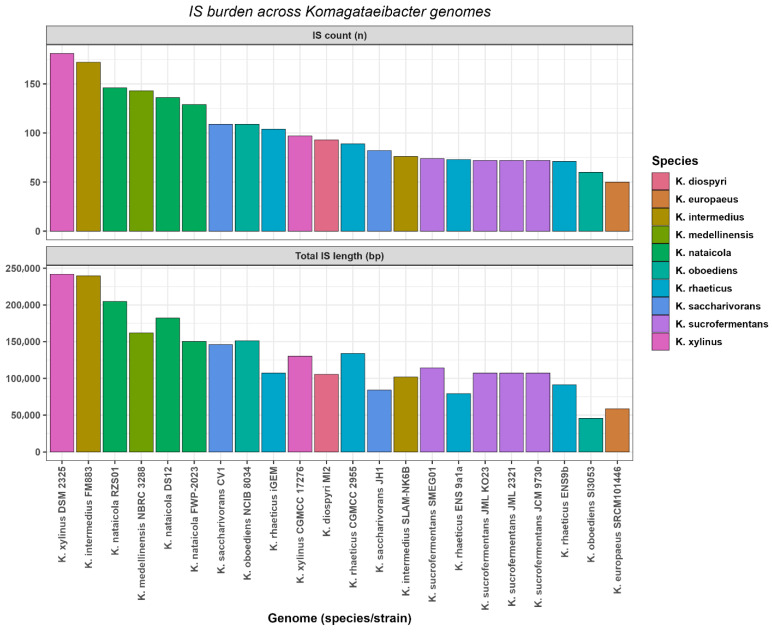
Insertion sequence burden across *Komagataeibacter* genomes.

Insertion sequence burden was summarized per genome using ISEScan predictions and is reported as element count and cumulative IS sequence length (Figure 6). Across the 18 genomes, IS counts ranged from 50 to 181 per genome, with a median of 91. Cumulative IS sequence length ranged from 45,537 bp to 242,015 bp per genome, with a median of 110,849 bp.

The highest IS counts and cumulative IS sequence lengths were observed in *K. xylinus* DSM 2325 and *K. intermedius* FM883. *K. xylinus* DSM 2325 contained 181 IS elements totaling 242,015 bp. *K. intermedius* FM883 contained 172 IS elements totaling 239,738 bp. The lowest IS counts and cumulative IS sequence lengths were observed in *K. europaeus* SRCM101446 and *K. oboediens* SI3053. *K. europaeus* SRCM101446 contained 50 IS elements totaling 58,588 bp. *K. oboediens* SI3053 contained 60 IS elements totaling 45,537 bp (Figure 6, Table S9). Assembly contiguity metrics did not show supported covariation with IS burden summaries, and the observed IS heterogeneity persisted after excluding the most fragmented assemblies (Table S9). Finally, the extent to which these mobilome summaries covaried with pangenome plasticity and cellulose regulation was evaluated using pairwise association tests.

### 3.8. Linkages Among Pangenome Plasticity, Mobilome Burden, and Cellulose Regulation

Pairwise associations among pangenome composition metrics, plasmid cluster counts, IS burden, and richness PCA coordinates were evaluated using Spearman correlations with multiple testing correction (Table S8). The largest effect sizes were observed among pangenome variables that share the same underlying denominators. Singleton count tracked singleton fraction, and accessory count tracked accessory fraction. For example, singleton count was strongly correlated with singleton fraction, with a Spearman rho of 0.994 and a q of 1.72 × 10^−19^. Accessory count was correlated with accessory fraction, with a Spearman rho of 0.910 and a q of 5.29 × 10^−8^. Total gene cluster count also showed positive associations with accessory-related metrics, including accessory content and variable fraction (Table S8).

In contrast, correlations linking richness coordinates to pangenome descriptors were weaker and did not remain significant after correction. The largest of these involved richness PC1 and singleton fraction, with Spearman rho of 0.415 and q of 0.141. Associations between plasmid cluster count and pangenome fractions were also weak in the subset with plasmid cluster counts. For example, accessory fraction and plasmid cluster count had a Spearman rho of 0.259 and a q of 0.710 (Table S8). The association between singleton fraction and richness PC1 was not supported under PGLS (Table S8). In the plasmid subset, accessory fraction showed a positive association with plasmid cluster count. This relationship was treated as exploratory given the reduced sample size and simplified plasmid summary variables. Overall, the observed correlation structure was driven by relationships among pangenome composition variables, whereas cross-module associations involving richness coordinates and plasmid cluster counts were not supported after multiple testing corrections at the current sampling depth.

## 4. Discussion

The dataset was curated to enable analyses that require a reliable resolution of structure and mobile-element architecture. Twenty-two *Komagataeibacter* assemblies annotated in RefSeq as closed or near closed were retrieved, and a non-clonal subset was retained for comparative mobilome analyses. According to RefSeq metadata, the retained genomes span fermented foods and plant-linked sources, such as vinegar and kombucha. Several assemblies lacked a linked primary publication in the associated records, which limited the strain-level interpretation of provenance and experimental context. Complete or near-complete assemblies are particularly valuable for *Komagataeibacter* because multiple plasmids can accompany the chromosome, and plasmid complements can differ sharply even among closely related strains [5,34]. In addition, both plasmids and repeated mobile elements are challenging to reconstruct and localize reliably from fragmented short-read assemblies, constraining comparative inference when draft genomes dominate [19,46]. These considerations motivated an explicit focus on complete assemblies as a foundation for interpreting plasmid repertoires and IS burden in a lineage-specific framework. A recent pangenome study of *Komagataeibacter* and *Novacetimonas* documented broad gene-content variability across the group and emphasized the importance of standardized genome resources for comparative inference [21]. This emphasis is aligned with recent strain-resolved complete-genome reports in *Komagataeibacter* that use closed assemblies to interpret cellulose-associated features and genome organization at a high resolution [20].

A single-copy core gene phylogeny provided a vertical inheritance scaffold for interpreting genome content variation within *Komagataeibacter*. This approach is consistent with phylogenomic frameworks used to resolve relationships within *Komagataeibacter* and closely related taxa, including lineages reassigned to *Novacetimonas* [4]. In the dataset, within-species clusters were well resolved, and several named species were separated by short internal branches. This topology enabled mobilome and accessory gene differences to be interpreted against a conserved genomic backbone rather than be attributed to deep divergence. The pangenome results reinforced the contrast between conserved and flexible components. A stable core fraction was observed across genomes, whereas the accessory fraction varied substantially. Similar patterns were reported in comparative genomics of *Komagataeibacter*, where a conserved backbone coexists with strain-specific flexible gene content that includes mobile DNA and functions linked to cellulose-associated traits [5,12]. Such noncore fractions are expected to be dynamic. They can be shaped by gene gain and loss through horizontal transfer and deletion, which can partially decouple gene-content variation from the core phylogeny over short evolutionary intervals [47]. For comparative analyses, core phylogenies are therefore routinely treated as the baseline for interpreting gene gain and loss processes in pangenome datasets [48]. These principles justify interpreting plasmid repertoires and other mobile features as contributors to non-core heterogeneity while retaining the core-genome phylogeny as the lineage reference.

Variation in cellulose synthesis and its proximal regulation was summarized using the richness module and its PCA projection. This projection provided a reduced description of differences in the local bcs context and nearby c-di-GMP signaling potential across lineages. Bacterial cellulose is a key industrial trait of *Komagataeibacter* because of the potential of high-value materials with broad application space [2,49]. At the genetic level, cellulose production is encoded by multi-gene bcs systems that differ in gene content and operon organization across bacteria, including multiple operon types described in comparative synthase frameworks [50]. In *Komagataeibacter*, complete genome studies have reported substantial variation in the number and organization of cellulose synthase operons, including multiple operons within a single chromosome [20,51]. Consistent with this literature, multi-copy signals were common for key targets in the present dataset, including bcsA. To enable comparable inference across genomes, a single representative bcs locus was used per genome and the analysis was complemented by architecture summaries. This strategy reduced ambiguity introduced by dispersed paralogs while retaining contrasts in local gene neighborhoods. The resulting PCA space showed broad dispersion, indicating that cellulose associated features and adjacent regulatory signals were not uniform across the genus. Mechanistically, the variation is plausible because cellulose synthase activity is directly controlled by c-di-GMP, which binds to the PilZ domain of BcsA and acts as an allosteric activator [8,52]. In addition, c-di-GMP signaling can be organized through local production and dedicated effector coupling, which supports the interpretation that differences in the local abundance of GGDEF and EAL domain proteins around bcs loci may reflect differences in regulatory coupling rather than only the presence or absence of structural synthase genes [53]. On this basis, the richness PCA may be viewed as a parsimonious summary of lineage-associated variation in cellulose-associated genomic features and their proximal regulatory context. This interpretation does not require strict congruence between core phylogeny and these regulation-adjacent features.

Plasmid sharing was concentrated in a subset of strains and resolved into a limited set of recurrent homology clusters rather than a broadly shared plasmid background. This pattern is consistent with plasmids forming a discontinuous component of the accessory genome, in which persistence and spread depend on host compatibility and ecological opportunity more than on vertical inheritance. The coexistence of species-restricted clusters and multi-species clusters is informative. Species-restricted clusters are compatible with stable maintenance within a narrow host range or restricted transfer. Multi-species clusters are compatible with broader host range backbones or the recurrent exchange of shared modules across species boundaries. A similar mixed structure has been described across bacterial systems, where plasmid evolution is frequently modular and shaped by recombination and horizontal transfer. In such systems, conserved plasmid families can coexist with mosaic assemblages that traverse taxonomic boundaries [22,54]. Within *Komagataeibacter*, comparative genomics has reported substantial strain-level variability in gene content and mobile element composition despite a conserved core genome structure [5,12]. Therefore, highly conserved clusters are consistent with shared plasmid backbones. More heterogeneous clusters are consistent with partial homology driven by module sharing rather than uniform backbone conservation.

In several genomes, the plasmid gene pool was dominated by mobility and maintenance-associated functions, which is consistent with architectures organized around replication, stability, and transfer modules. The non-mobility component varied among strains and included predicted categories plausibly linked to stress response and defense. This configuration aligns with a general principle in bacterial genomics. Plasmids can couple transfer capacity with accessory traits that are conditionally beneficial, thereby shaping strain-specific performance under particular environments [22]. This perspective is relevant to acetic acid bacteria used in fermentation, where industrially important phenotypes include tolerance to acetic acid, ethanol, and oxidative stress. Complete genome and comparative studies in *Acetobacter pasteurianus* have linked high-tolerance phenotypes to genomic differences and have explicitly reported substantial plasmid sequence content alongside chromosomes in tolerant strains [55,56,57]. Accordingly, plasmid repertoires can be regarded as a plausible reservoir of strain-specific adaptive potential in *Komagataeibacter*, while the contribution of individual plasmid-encoded loci to defined fermentation-related traits remains to be established through targeted experimental validation.

In contrast to plasmids, VIBRANT detected only a small number of prophage-associated regions per genome, and these regions were treated as fragments rather than validated intact and inducible prophages. This pattern is consistent with genome-scale observations across bacteria, where integrated phage-derived sequences are common but often show decay through mutation, rearrangement, and deletion, yielding cryptic or defective prophage remnants that differ among closely related strains [58]. Progressive degradation is also expected to reduce the cross-genome conservation of prophage repertoires, even when strains share a conserved core backbone [59]. Alongside these prophage fragments, insertion sequences provided a distinct axis of mobilome heterogeneity. Substantial variation in IS counts and cumulative IS length indicates that transposition-associated sequence burden differs across *Komagataeibacter* genomes. Insertion sequences are recognized drivers of genome plasticity. They can disrupt coding sequences, alter local gene expression, and promote rearrangements, deletions, and duplications [60]. These mechanisms can generate strong strain-specific genomic signatures even when identifiable cargo categories are not enriched near IS elements under strict screening criteria. IS burden differences are consistent with unequal transposition-associated sequence content across genomes, but the timing of IS accumulation cannot be resolved without longitudinal or population-level data. In this dataset, prophage-associated regions were sparse and appeared to be largely genome-specific, which is consistent with the progressive degradation and limited cross-genome conservation of prophage remnants. In parallel, insertion sequences showed pronounced variation in burden and therefore support a role for transposition-driven structural remodeling rather than the predictable enrichment of transferable functional modules. These mobilome components operate on different timescales and via different mechanisms than plasmid-mediated gene flux. This distinction is important when cross-module linkages are evaluated. Simple correlations between mobilome burden and functional summaries can be weak or unstable when traits are shaped by both shared ancestry and episodic gene gain and loss. Accordingly, the linkage analysis is best interpreted considering its statistical and phylogenetic limitations.

The cross-module linkage analysis was primarily informative for identifying expected dependencies among derived pangenome descriptors. Singleton counts tracked singleton fractions and accessory counts tracked accessory fractions because these fractions are calculated directly from the corresponding counts and the total number of gene clusters. After multiple testing correction, no statistically robust associations were detected between plasmid cluster abundance or the richness PCA coordinates and pangenome plasticity metrics. This outcome does not exclude biologically meaningful relationships. It indicates that such relationships were not resolved under the current sampling depth, summary variables, and modeling assumptions.

Several limitations should therefore be emphasized. First, statistical power was constrained by the modest number of complete genomes available for analysis. Power was further reduced for plasmid-linked variables because plasmid cluster counts were available for only a subset of genomes. Second, this study was based on in silico inference. Mobility classes, prophage boundaries, and functional annotations were not validated experimentally. Third, key predictors were necessarily simplified. A single plasmid cluster count does not distinguish conserved backbones from module sharing assemblages, and it does not capture transfer potential. Fourth, linkage in bacterial comparative genomics is affected by shared ancestry. Closely related strains are not independent observations, which can obscure or inflate associations when phylogeny is not modeled explicitly. Phylogeny-aware association frameworks have been developed to mitigate this problem in microbial settings [61]. In addition, several taxa are represented by single genomes, so apparent species-specific patterns should be considered preliminary pending broader complete-genome sampling. Finally, different mobilome components reflect different mechanisms and timescales of genome change. IS activity, prophage decay, and plasmid turnover need not covary linearly with gene content summaries across strains. Within these constraints, the present results support conservative conclusions. Mobilome heterogeneity was evident across *Komagataeibacter* genomes, but simple cross-module correlations were not sufficient to connect mobilome summaries to cellulose-associated signatures or pangenome plasticity in a statistically robust manner. Future work will benefit from expanded complete genome sampling, phylogeny-aware association models, and predictors that directly represent transfer capacity and structural remodeling.

## 5. Conclusions

This study established a complete-genome comparative framework for integrating lineage structure, cellulose locus context, and mobilome variation across *Komagataeibacter*. Complete assemblies reduced ambiguity in plasmid inventories, prophage region boundaries, and insertion sequence burden. Core genome phylogenomics provided the lineage scaffold, and pangenome analysis supported a conserved core component alongside substantial genome-specific variability in the non-core fraction. Variations in cellulose-locus neighborhood features and nearby c-di-GMP-associated annotations were summarized using a targeted feature set, with locus anchoring and architecture summaries used to handle multi-copy targets.

Mobilome profiles were strongly strain-dependent. Plasmid sharing was concentrated in a subset of strains and resolved into recurrent homology clusters that included both species-restricted and multi-species groups. Independent Mash validation supported conserved backbone relationships for several clusters, whereas at least one cluster was consistent with more heterogeneous module sharing. Prophage-associated regions were detected across genomes but were sparse in number and were dominated by medium-length fragments, which is consistent with prophage decay. Insertion sequence burden varied widely, indicating that transposition-associated DNA constitutes a major and uneven component of mobilome heterogeneity within the genus. Cross-module linkage analysis did not yield statistically robust associations beyond expected dependencies among derived pangenome descriptors, which supports conservative interpretation at the current sampling depth.

These resources provide a curated reference for comparative work in *Komagataeibacter*. The complete genome backbone, strain-resolved plasmid clusters with validation, and mobilome burden summaries can support candidate selection for experimental follow up and hypothesis-driven studies on traits relevant to bacterial cellulose production and robustness. As additional complete genomes accumulate, the same framework can be extended with phylogeny-aware association models and more specific mobilome predictors to test how cellulose locus architecture, regulatory organization, and mobile DNA jointly shape strain diversity in biotechnology and fermentation-associated environments.

## Figures and Tables

**Table 1:** *Komagataeibacter* genomes included in this study and associated metadata.

Strain	Taxonomy Id	Accession	Size	Submission Date	Source	Reference
*K. medellinensis* NBRC 3288	634177	GCF_000182745.2	3,513,191	25 September 2011	Vinegar	[33]
*K. nataicola* RZS01	265960	GCF_002009295.1	3,760,301	2 March 2017	Rotten apple	[34]
*K. europaeus* SRCM101446	33995	GCF_002173515.1	3,797,909	6 June 2017	NA	unpublished
*K. saccharivorans* CV1	265959	GCF_003546645.1	3,768,311	11 September 2018	Vinegar	NA
*K. xylinus* DSM 2325	28448	GCF_004006375.1	3,727,795	9 January 2019	NA	[35]
*K. saccharivorans* JH1	265959	GCF_004348195.1	3,727,857	13 March 2019	Fruit fly	[36]
*K. xylinus* CGMCC 17276	28448	GCF_009834365.1	3,983,026	8 January 2020	Green jujube	[37]
*K. oboediens* SI3053	65958	GCF_019052775.1	3,666,777	28 June 2021	Apple cider vinegar production	[38]
*K. oboediens* NCIB 8034	65958	GCF_029229465.1	4,059,958	17 March 2023	Kombucha tea culture	NA
*K. nataicola* DS12	265960	GCF_029229505.1	3,916,688	17 March 2023	Kombucha tea culture	NA
*K. nataicola* FWP-2023	265960	GCF_032075085.1	3,767,936	27 September 2023	NA	[39]
*K. rhaeticus* CGMCC 2955	215221	GCF_034063025.1	3,563,316	4 December 2023	Vinegar	[40]
*K. sucrofermentans* JCM 9730	1307942	GCF_040581405.1	3,437,745	11 July 2024	Black cherry	[41]
*K. diospyri* MI2	1932662	GCF_043995075.1	3,885,945	30 October 2024	Ripen Sapodilla fruit	[42]
*K. sucrofermentans* SMEG01	1053551	GCF_047795655.1	3,442,337	18 February 2025	Apple	[20]
*K. intermedius* SLAM-NK6B	66229	GCF_049202445.1	3,661,158	4 April 2025	Kombucha	[43]
*K. intermedius* FM883	66229	GCF_052445895.1	4,235,196	4 September 2025	Kombucha tea	[44]
*K. rhaeticus* iGEM	215221	GCF_900086575.1	3,867,346	1 June 2016	Kombucha tea Scoby	[45]

Footnote: “NA” indicates that the corresponding field was not provided in the associated public genome record or could not be reliably recovered from linked metadata at the time of curation. “Unpublished” indicates that no peer-reviewed publication was found in the assembly record in RefSeq.

## Data Availability

The original contributions presented in this study are included in the article/Supplementary Material. Further inquiries can be directed to the corresponding author.

## Supplementary Materials

The following supporting information can be downloaded at: https://www.mdpi.com/article/10.3390/microorganisms14030653/s1, Figure S1. Plasmid-associated cargo categories across *Komagataeibacter* genomes. Horizontal stacked bars show the number of unique plasmid-associated genes per strain assigned to six putative biotechnology- and fitness-relevant categories (defense/stability, metal tolerance, acid/pH, osmotic stress, EPS/cell envelope, and oxidative stress). Counts are based on predicted functional annotations and are presented as comparative descriptors of plasmid gene-content variation across strains, not as demonstrated phenotypic effects; Figure S2. Length distribution of predicted prophage regions in Komagataeibacter. Histogram showing the size distribution of prophage-associated regions predicted by VIBRANT across the analyzed genomes; Table S1. Genome Lengths and Plasmid contents of Komagataeibacter genome sequences; Table S2. Pangenome plasticity metrics across Komagataeibacter genomes; Table S3. Plasmid accessions to plasmid homology clusters across Komagataeibacter genomes; Table S4. Mash-based validation of plasmid homology clusters; Table S5. Per-genome distribution of plasmid-associated biotech cargo categories (unique gene count); Table S6. (A). Region Inventory of Putative Prophages, (B). Prophage Caller Comparison; Table S7. Best cross-genome mash match for each VIBRANT-predicted prophage region; Table S8. Spearman correlations linking pangenome plasticity, plasmid repertoire, IS burden, and cellulose/regulation richness; Table S9. ISEScan derived insertion sequence burden summaries and IS calls across Komagataeibacter genomes; Table S10. MOB-suite plasmid mobility predictions and relaxase-type annotations across Komagataeibacter genomes.
