# Supplementary material for: Comparative Analysis of Lineage Structure, Cellulose Locus Context, and Mobilome Diversity Across Complete Komagataeibacter Genomes
Source: Microorganisms. 2026 Mar 13;14(3):653. doi: 10.3390/microorganisms14030653 (PMC13028821; doi:10.3390/microorganisms14030653)
Supplement: Supplementary file 1 [file microorganisms-14-00653-s001.zip › Supplementary_Figures.pdf]

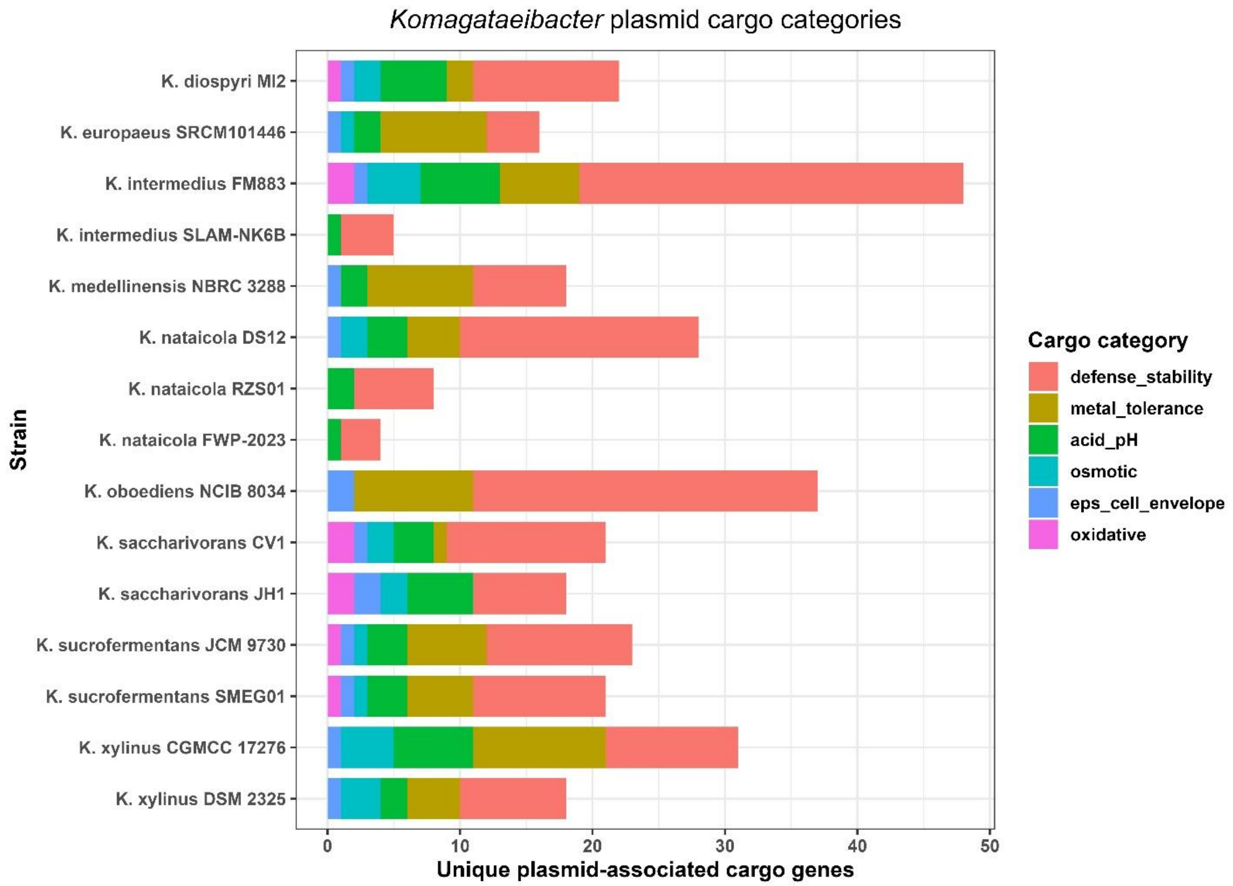

Figure S1. Plasmid-associated cargo categories across *Komagataeibacter* genomes. Horizontal stacked bars show the number of unique plasmid-associated genes per strain assigned to six putative biotechnology- and fitness-relevant categories (defense/stability, metal tolerance, acid/pH, osmotic stress, EPS/cell envelope, and oxidative stress). Counts are based on predicted functional annotations and are presented as comparative descriptors of plasmid gene-content variation across strains, not as demonstrated phenotypic effects.

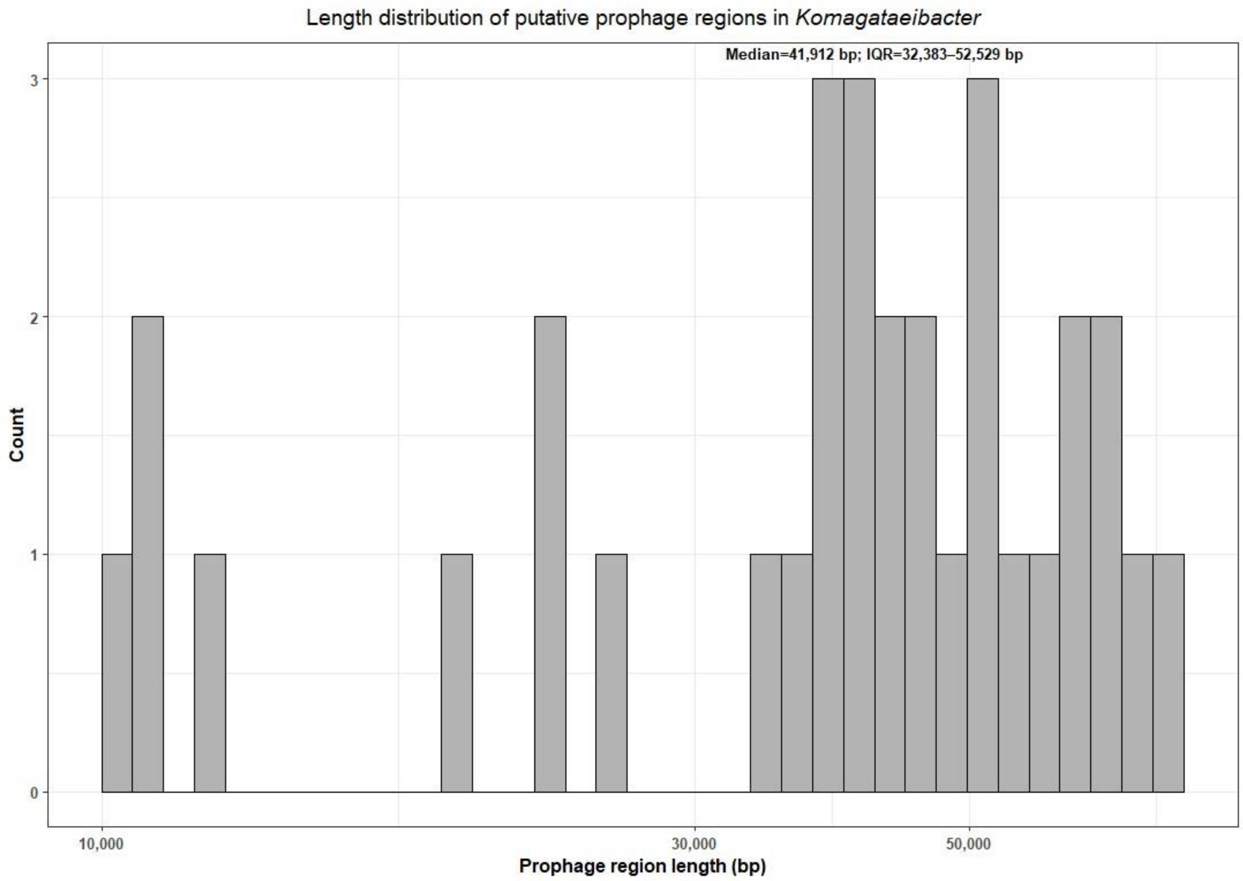

Figure S2. Length distribution of predicted prophage regions in Komagataeibacter.

Histogram showing the size distribution of prophage-associated regions predicted by VIBRANT across the analyzed genomes.
